# Smart and Efficient Synthesis of Cyclic Poly(*N*-isopropylacrylamide)s by Ring Expansion RAFT (RE-RAFT) Polymerization and Analysis of Their Unique Temperature-Responsive Properties

**DOI:** 10.3390/molecules29225392

**Published:** 2024-11-15

**Authors:** Jin Motoyanagi, Kenichi Bessho, Masahiko Minoda

**Affiliations:** Faculty of Molecular Chemistry and Engineering, Graduate School of Science and Technology, Kyoto Institute of Technology, Matsugasaki, Sakyo-ku, Kyoto 606-8585, Japan

**Keywords:** RAFT polymerization, cyclic polymer, poly(*N*-isopropylacrylamide), ring-expansion RAFT polymerization, cyclic trithiocarbonate derivative, thermo-responsive property

## Abstract

Cyclic polymers have many interesting properties compared to their linear analogs, but there are very few examples of their synthesis. This is because most cyclic polymers have been synthesized by stepwise processes, including synthesizing homo- or hetero-telechelic end-functionalized precursor polymers and consecutive intramolecularly coupling of both ends of the polymers. This requires a complicated synthesis, and the product yields are very low because the target cyclic polymers are usually synthesized under highly dilute conditions, consequently, making it difficult to systematically analyze the properties of cyclic polymers. In the present research, we have synthesized cyclic polymers using a ring expansion polymerization method. Particularly, the ring expansion RAFT polymerization (RE-RAFT polymerization) that we have developed using a cyclic chain transfer agent is a smart method that can synthesize cyclic polymers very efficiently. In this paper, we successfully synthesized cyclic-poly(*N*-isopropylacrylamide), which is widely known as a thermo-responsive polymer, by RE-RAFT polymerization. Furthermore, we have compared the thermo-responsive properties of the cyclic-poly(*N*-isopropylacrylamide)s with those of their linear analogs.

## 1. Introduction

In recent years, cyclic polymers with no chain ends have exhibited many interesting properties compared to their linear analogs, such as distinguished glass transition temperature, viscosity, diffusion behavior, and hydrodynamic volume [1,2,3,4,5]. However, the synthesis of cyclic polymers with external stimuli-responsive polymers as the backbone moiety has been very limited because the synthesis is only possible through stepwise methods, resulting in very low yields [6,7]. The present study focuses on the thermo-responsive polymer among external stimulus-responsive polymers and aims to develop an efficient synthetic method for cyclic temperature-responsive polymers. In previous research, linear and cyclic poly(*N*-isopropylacrylamide) (polyNIPAM), which is one of the representative thermo-responsive polymers, have been demonstrated to display disparate aggregation and phase transition behaviors [8,9,10]. Because the phase transition temperature of polyNIPAM is proximate to body temperature [11,12], polyNIPAM and its derivatives have been well recognized as promising materials in biomedical and biotechnology fields [13,14,15]. Moreover, Tezuka has demonstrated that the thermal stability of self-assembling polymeric micelles is significantly influenced by topological factors [16,17,18]. The designed amphiphilic linear triblock copolymers and their cyclization products were observed to self-assemble into flower-like micelles. However, the cloud point (CP) of the cyclic polymers was found to increase by over 40 °C in comparison to the linear polymers, despite no significant change in chemical composition [17]. Thus, cyclic polymers exhibit distinctive properties that diverge from those of linear counterparts in response to external stimuli, and they are highly anticipated as new functional materials.

Over the past few decades, numerous researchers have endeavored to synthesize cyclic polymers. The most prevalent synthetic methodology is the end coupling method. First, homo- or hetero-telechelic polymers are synthesized by introducing reactive groups at both ends of a linear polymer using living polymerization, and then cyclic polymers are synthesized by intramolecular coupling of the terminal functional groups [3]. Therefore, this synthetic method requires a multi-step synthetic process and the coupling reactions usually need to be carried out under highly dilute conditions to favor intramolecular reactions, resulting in very low product yields. It was therefore a cumbersome task to systematically synthesize cyclic polymers and investigate the correlation between their structure and properties. On the other hand, ring-expansion polymerization (REP) has been developed as an alternative method for the synthesis of cyclic polymers. REP is superior to the end-coupling methods mentioned above because it is a smarter, more straightforward synthetic approach and allows for the yielding of large amounts of cyclic polymers [19,20]. In REP, the utilization of designed cyclic initiators that can generate a couple of active sites within a molecule allows the synthesis of cyclic polymers; therefore, the cyclic topology is maintained throughout the polymerization by successive insertion of monomers. The earliest examples of REP work include the synthesis of cyclic polycaprolactones using cyclic alkoxytin derivatives as initiators by Kricheldorf [21]. Additionally, Grubbs has demonstrated the successful synthesis of cyclic polyolefins by ring-opening metathesis polymerization employing designed cyclic carbene–ruthenium complexes as initiators [22,23]. In addition, Ouchi used a cyclic hemiacetal ester compound as an initiator for living cationic polymerization and succeeded in the synthesis of cyclic polyvinyl ethers [24,25]. Moreover, the synthesis of cyclic polylactides by ring-opening polymerization of lactides using nitrogen-containing heterocyclic carbenes as activators has also been reported [26]. Thus, several methods for the synthesis of cyclic polymers using REP have been reported, but the types of monomers that can be applied are limited, and there are very few examples of the synthesis of cyclic polymers from commonly used vinyl monomers.

**Scheme 1:**
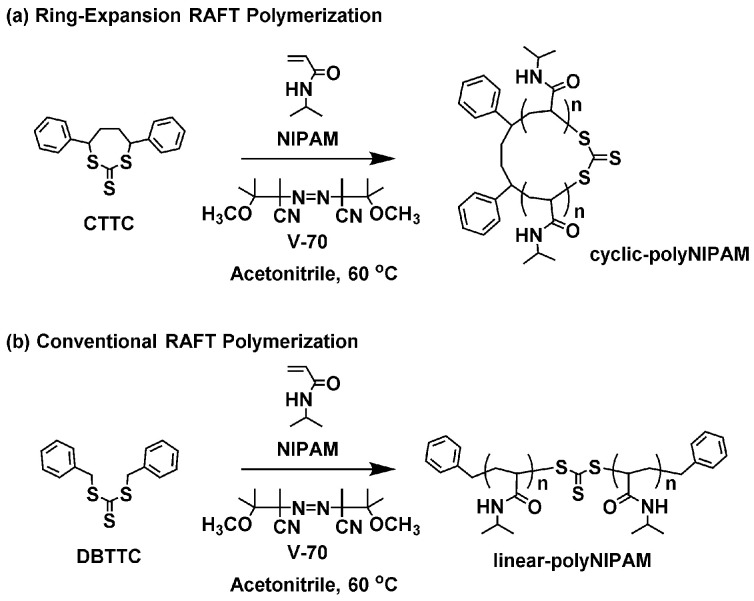
RAFT polymerization of NIPAM (**a**) with CTTC as a cyclic RAFT agent and (**b**) with DBTTC as an acyclic RAFT agent.

To establish a more versatile strategy for the synthesis of cyclic polymers using REP, we focused on reversible addition-fragmentation chain transfer (RAFT) polymerization [27,28,29,30,31,32], which is applicable to a wide range of vinyl monomers such as (meth)acrylates, (meth)acrylamides, and styrene derivatives and has the potential to control molecular weight and repeating unit arrangement (e.g., block polymer structures). In RAFT polymerization, monomers are inserted between the growing ends of the polymer chain and the RAFT agent residues. Therefore, we have conceived of an efficient synthesis of cyclic polymers by ring-expansion RAFT (RE-RAFT) polymerization using a cyclic RAFT agent with two potential reaction sites in the molecule. RE-RAFT polymerization using cyclic chain transfer agents has been previously reported by Pan, but under very limited conditions, i.e., under γ-irradiation with ^60^Co and at −30 °C [33]. We have already succeeded in the efficient synthesis of cyclic-poly(*tert*-butyl acrylate) for the first time by performing RE-RAFT polymerization of *tert*-butyl acrylate (TBA) using cyclic trithiocarbonate (4,7-diphenyl-[1,3]dithiepane-2-thione, hereinafter referred to as CTTC) as a RAFT agent [34]. The structural analyses of the obtained polymers using size exclusion chromatography (SEC), ^1^H NMR, and matrix-assisted laser desorption ionization-time of flight mass spectrometry (MALDI-TOF-MS) revealed the formation of cyclic-polyTBA. Furthermore, we found that the “ring-fusion” of the generated cyclic polymers due to the cross-propagation occurred during the RE-RAFT polymerization, resulting in the production of a mixture of cyclic polymers of various sizes [34]. In this study, with the aim of synthesizing functional cyclic polymers, we investigated the synthesis of cyclic-polyNIPAM (Scheme 1a) by the RE-RAFT polymerization of *N*-isopropylacrylamide. The major difference between this study and the synthesis of cyclic-polyNIPAMs in previous research [8,9,10] is that the synthesis of cyclic-polyNIPAM by RE-RAFT polymerization can be performed in a simple one-pot synthesis with high yield. The obtained polymers were characterized in detail by ^1^H NMR, SEC, and MALDI-TOF-MS. Furthermore, to investigate the unique thermo-responsive properties of the cyclic-polyNIPAMs, we synthesized cyclic-polyNIPAMs with different molecular weights and compared their thermo-responsive properties with those of the linear analogs. This RE-RAFT polymerization is a smart synthetic method that can efficiently obtain cyclic polymers by simply adding a cyclic RAFT agent to a conventional free radical polymerization system. For example, it is possible to synthesize multi-component cyclic polymers by copolymerization of *N*-isopropylacrylamide with other acrylamide monomers, so it is expected that it can be applied to the control of thermo-responsive properties.

## 2. Results

### 2.1. Ring-Expansion RAFT (RE-RAFT) Polymerization of NIPAM with CTTC

**Figure 1:**
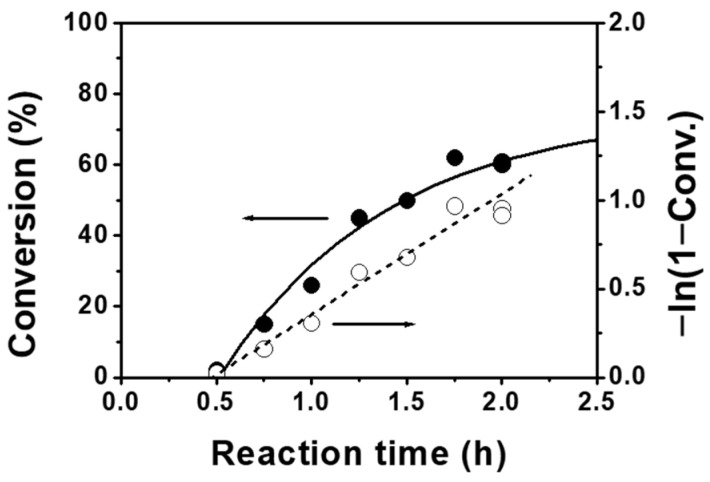
A time-conversion curve (black circles) and pseudo-first-order kinetic plot (white circles) for the polymerization of NIPAM. Polymerization was conducted with CTTC/V-70 in acetonitrile at 60 °C ([NIPAM]_0_/[CTTC]_0_/[V-70]_0_ = 200/1/0.4, [NIPAM]_0_ = 20 wt%).

**Figure 2:**
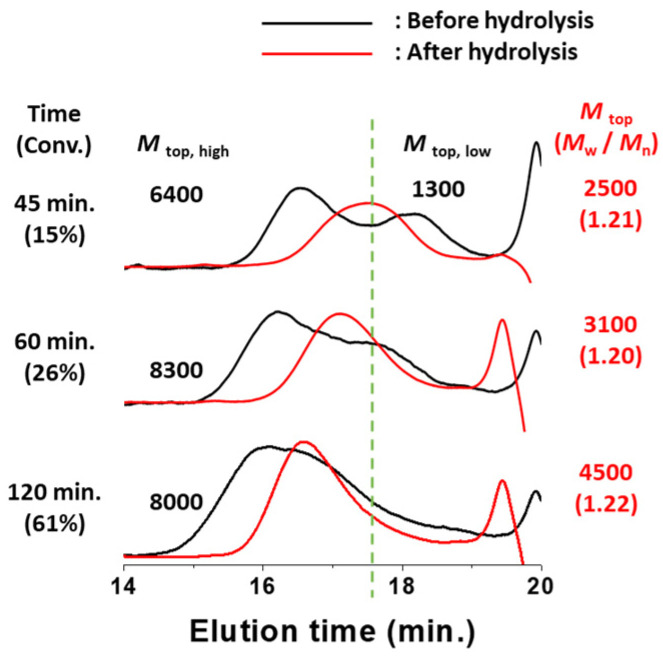
SEC curves of polyNIPAMs measured in THF. Polymerization was conducted with CTTC/V-70 in acetonitrile at 60 °C ([NIPAM]_0_/[CTTC]_0_/[V-70]_0_ = 200/1/0.4, [NIPAM]_0_ = 20 wt%) (Black line). SEC curves of the cleaved segment polyNIPAM from the generated polymers by RE-RAFT polymerization of NIPAM with CTTC obtained at various NIPAM conversions (Red line).

**Scheme 2:**
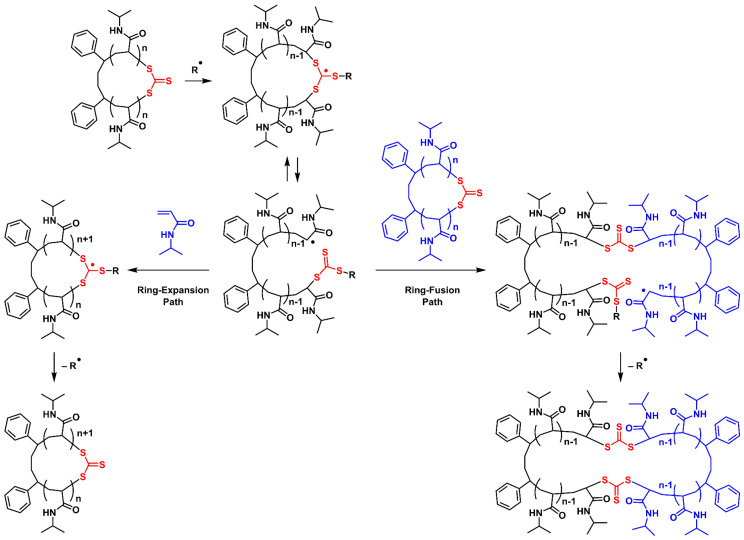
Schematic of ring-expansion RAFT polymerization of NIPAM with a cyclic RAFT reagent CTTC containing “ring-expansion paths” and “ring-fusion paths”.

**Scheme 3:**
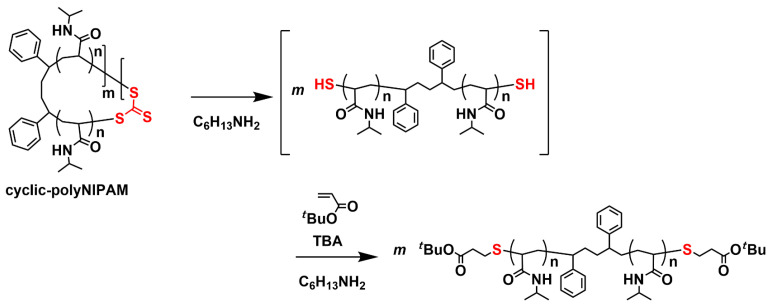
The hydrolysis of the cyclic-polyNIPAM with hexylamine followed by the Michael addition reaction with TBA.

We investigated RE-RAFT polymerization of NIPAM at 60 °C in acetonitrile using 2,2′-azobis(4-methoxy-2,4-dimethylvaleronitrile) (V-70) as a radical initiator and CTTC as a cyclic RAFT reagent. ([NIPAM]_0_/[CTTC]_0_/[V-70]_0_ = 200/1/0.4, [NIPAM]_0_ = 20 wt%) (Scheme 1a). In Figure 1, the time-conversion curve obtained for the polymerization of NIPAM showed an induction phase of approximately 0.5 h, reaching a conversion of about 70% within 2 h. After the induction phase, the pseudo-first-order kinetic plot of NIPAM polymerization showed a linear relationship, indicating that the concentration of the growing active species remained constant throughout the polymerization, suggesting that a controlled polymerization process occurred. However, SEC analysis showed peculiar polymerization behavior, similar to the previously reported RE-RAFT polymerization of TBA [34]. In fact, from the very early stages of polymerization (conv.~15%), the chromatogram showed bimodal peaks, including a new peak appearing in the high molecular weight region (Figure 2, black line) (Table 1). This phenomenon is due to the fact that two pathways occur simultaneously in this RE-RAFT polymerization [23,34]: the “ring-expansion pathway”, in which cyclic polymers are synthesized by ring expansion polymerization with monomer insertion into the cyclic species, and the “ring-fusion pathway”, by intermolecular chain transfer reactions between the formed cyclic polymers containing one or more trithiocarbonate moieties (Scheme 2). The latter path resulted in generating higher molecular weight cyclic polymers. Considering the reaction mechanism of the “ring-fusion pathway”, the resulting high-molecular-weight polymers are expected to have a ring structure in which multiple polyNIPAM segments are linked to each other via multiple trithiocarbonate (TC) moieties. Therefore, cleavage of the TC moieties yields individual polyNIPAM segments, and a detailed investigation of the resulting polyNIPAM segment polymers provides useful information about the polymerization behavior of the RE-RAFT polymerization of NIPAM. As previously reported [34], the TC moieties connecting the polyNIPAM segments are expected to be hydrolyzed by primary amines to generate thiol end groups, so by adding an excess amount of TBA and performing a Michael addition reaction to prevent the formation of inter- and intramolecular disulfide bonds, polyNIPAM polymers capped at both ends with TBA residues can be obtained. The polymers obtained by RE-RAFT polymerization were hydrolyzed with hexylamine in THF followed by the reaction with TBA (Scheme 3). After 1 h of reaction at room temperature, the UV–vis spectrum of the reaction mixture showed the disappearance of the 310 nm peak assignable to the TC moieties, indicating that the quantitative hydrolysis had progressed. The obtained polyNIPAM from the cleavage reaction was then characterized by SEC to study the growth of polyNIPAM segments during RE-RAFT polymerization (Figure 2, red line). Regardless of the reaction time, the SEC curves showed a unimodal peak, and as monomer conversion increased, the peak top shifted to the high molecular weight region, while the molecular weight distribution (*M*_w_/*M*_n_) remained relatively narrow, around 1.2. The *M*_n_ values calculated from the SEC curves were found to be smaller than the theoretical molecular weights. This discrepancy is likely due to the interaction between the SEC column and polyNIPAM caused by the use of THF as the eluent in the SEC measurement. These results indicate that the polymerization of NIPAM in the RE-RAFT polymerization conditions using the cyclic RAFT reagent CTTC proceeded in a controlled manner, producing cyclic-polyNIPAM consisting of multiple polyNIPAM segments with uniform chain lengths linked with TC groups.

### 2.2. Characterization of the Obtained Polymers by RE-RAFT Polymerization with CTTC

**Figure 3:**
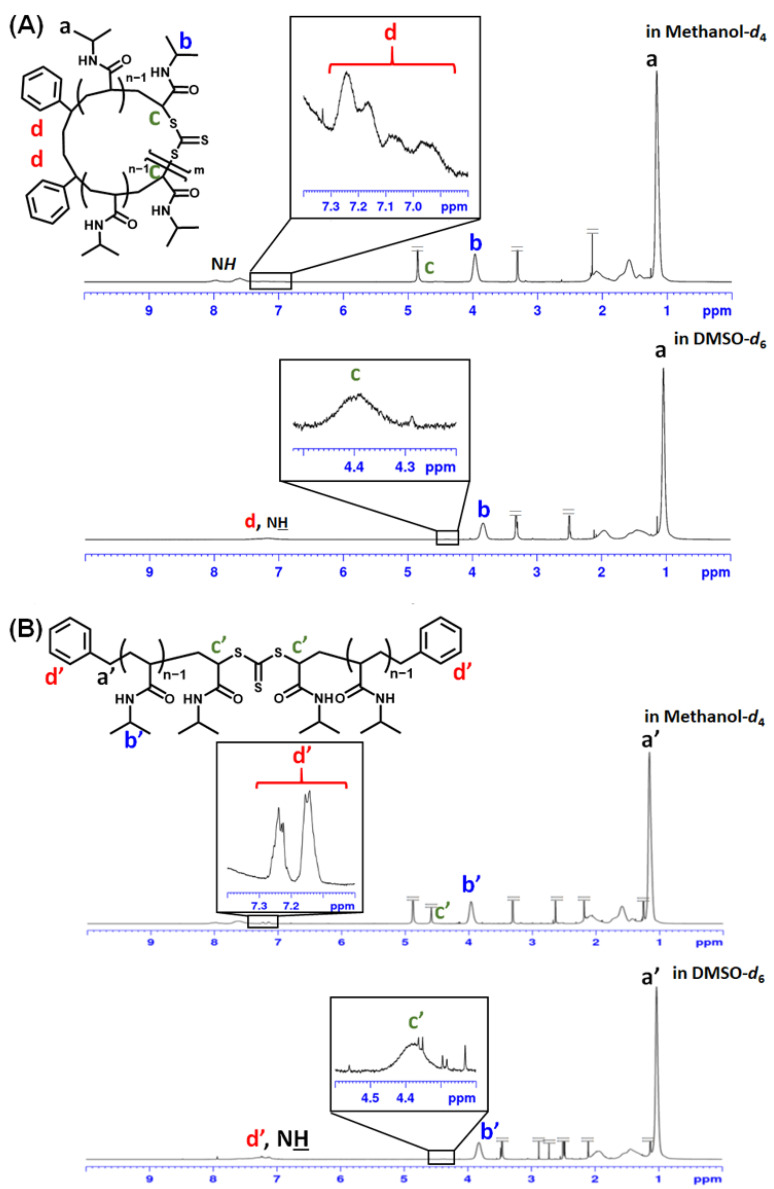
^1^H NMR spectra of (**A**) cyclic-polyNIPAM_122_ by CTTC/V-70 and (**B**) linear-polyNIPAM_146_ by DBTTC/V-70 in methanol-*d*_4_ and DMSO-*d*_6_.

After reprecipitation of the obtained cyclic-polyNIPAM (good solvent; acetone, poor solvent; hexane), the structure of the isolated cyclic-polyNIPAM_122_ (under the same polymerization conditions as in Figure 1 and Figure 2, NIPAM conversion = 61%) was analyzed in detail by ^1^H NMR spectroscopy (Figure 3A). As a reference polymer, linear-polyNIPAM_146_ was synthesized separately by conventional RAFT polymerization using *S*,*S*-dibenzyl trithiocarbonate (DBTTC) as an acyclic RAFT reagent at 60 °C in acetonitrile ([NIPAM]_0_/[DBTTC]_0_/[V-70]_0_ = 200/1/0.4, [NIPAM]_0_ = 20 wt%, NIPAM conversion = 73%, linear-polyNIPAM: *M*_n_ = 6600, *M*_w_/*M*_n_ = 1.21; estimated by polystyrene calibrated SEC in THF) (Scheme 1b). The ^1^H NMR spectra of the linear-polyNIPAM_146_ are shown in Figure 3B. The key protons for the structural analysis are the phenyl proton peak (peaks d′) arising from the end of the linear-polyNIPAM_146_, the pair of methine protons (peak c′) adjacent to the TC moieties, and the methine peak of the pendant isopropyl groups (peak b′). In DMSO-*d*_6_, peak c′ was clearly observed at 4.3–4.5 ppm, but peaks d′ at 6.9–7.3 ppm overlapped with the NH proton of the amide group, so their integral ratio cannot be directly estimated. On the other hand, in methanol-*d*_4_, peaks d′ appeared at 6.9–7.3 ppm, but peak c′ overlapped with the residual solvent, so a direct comparison of their integral ratio is not possible. Therefore, the isopropyl methine protons (peak b′), which was clearly observed at 3.8–4.1 ppm in both deuterated solvents, was used as the standard peak, and the integral ratio of peak d′ to peak c′ was estimated from the ratio of peak c′/peak b′ in DMSO-*d*_6_ and the ratio of peak d′/peak b′ in methanol-*d*_4_. As a result, the peak intensity ratio of the terminal phenyl protons (peak d′) and the methine protons adjacent to the TC group (peak c′) was determined to be 10.0:1.96, which is in good agreement with the expected telechelic structure (Figure 3B). Based on the peak intensity ratio of peak d′/peak b′ in methanol-*d*_4_, the number-average degree of polymerization (*DP*_n_) of the linear-polyNIPAM_146_ was evaluated to be 147. This *DP*_n_ value was in good agreement with the theoretical value of 146, which was calculated based on the feed molar ratio of NIPAM to DBTTC and the monomer conversion. By comparison with the ^1^H NMR data of the linear-polyNIPAM_146_, we identified the chemical structure of the obtained polyNIPAM based on RE-RAFT polymerization (Figure 3A). The phenyl proton peaks d derived from the CTTC residue appeared at 6.9–7.4 ppm in methanol-*d*_4_ and the characteristic peak c at 4.4 ppm of the methine protons adjacent to the TC moieties was also observed in DMSO-*d*_6_. The ratio of the resonance intensities of the phenyl protons versus methine protons [Figure 3A, peaks d and peak c, respectively] was 10.0:1.96, which indicates that NIPAM monomers were inserted between the C–S bond of the TC moiety and the benzyl carbon (–S–C(=S) –***S***–***C***H(Ph)CH_2_CH_2_–) in the initiation process of the RE-RAFT polymerization and consecutive insertions during the propagation. This result indicates that the molecular structure thoroughly retained the cyclic structure of CTTC and the resulting polyNIPAMs consist of a cyclic structure without termini. Furthermore, *DP*_n_ of the polyNIPAM segments was evaluated to be 137 from the intensity ratio of the peak b and peak c. This *DP*_n_ value is nearly identical to the theoretical one (Theoretical *DP*_n_ = [NIPAM]_0_/[CTTC]_0_ × (conversion of NIPAM)). If acyclic polyNIPAM were included in the product polymers, these ratios (peak area ratios of peak d/peak c and peak b/peak c) would deviate from the theoretical values, clearly indicating that the target cyclic polyNIPAM was formed based on RE-RAFT polymerization.

**Figure 4:**
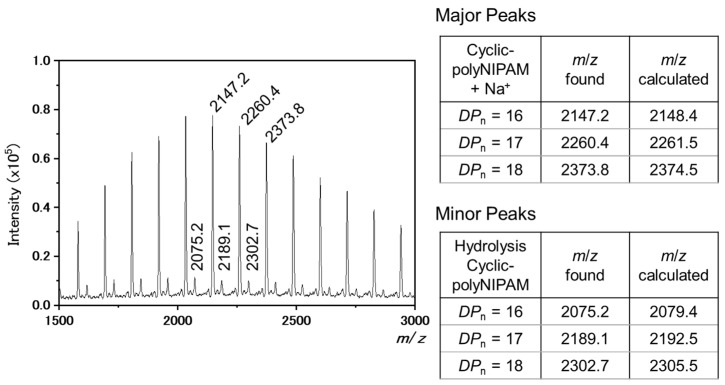
MALDI-TOF MS of the cyclic-polyNIPAM_30_ (matrix: 9-nitroanthracene; cationizing agent: sodium trifluoroacetate).

To further verify that the polyNIPAM synthesized by the RE-RAFT polymerization has a cyclic structure, MALDI-TOF-MS measurements (matrix: 9-nitroanthracene, cationizing agent: sodium trifluoroacetate) were performed. With the aim of obtaining data with higher resolution, we employed the polymer sample produced at the early stage of the RE-RAFT polymerization. Figure 4 shows the MALDI-TOF-MS of polyNIPAM (cyclic-polyNIPAM_30_) obtained at Conv. = 15% under the same polymerization conditions as in Figure 1 and Figure 2. Figure 4 depicts a series of the peaks attributed to the cyclic-polyNIPAM of various *DP*_n_ along with a summary comparing the observed and calculated *m*/*z* values. The calculated *m*/*z* values were based on the cyclic-polyNIPAM having a CTTC moiety and repeating NIPAM units, ionized with Na^+^ [CTTC-(NIPAM)*_n_* + Na]^+^ (*M*_n_ = 316 + (113 × *n*) + 23). The observed and calculated *m*/*z* values are nearly identical, suggesting that the obtained polyNIPAMs possess no terminal structure and consist only of a cyclic structure. And the minor series of peaks are probably attributable to the structure in which the trithiocarbonate group in cyclic-polyNIPAM is hydrolyzed to thiol groups under ionization conditions (calculated *m*/*z* values; *M*_n_ = 316 + (113 × *n*) − 44).

### 2.3. Temperature-Responsive Properties of the Cyclic-polyNIPAM and Linear-polyNIPAM

One of the fundamental physical properties of temperature-responsive water-soluble polymers is the thermal phase transition at a specific temperature that is specific to the polymer structure, expressed as the lower critical solution temperature (LCST) and/or cloud point (CP) temperature. Liu et al. compared the thermal phase transition temperatures of cyclic and linear polyNIPAM [8]. In their study, they measured the LCST and CP temperatures of cyclic and linear polyNIPAM in aqueous solution using a temperature-dependent turbidity measurement method. Here, the LCST and CP temperatures are defined as the temperatures at which the transmittance decreases by 1% and 50%, respectively. They found that the LCST and CP temperature of the linear polymer were 34 °C and 37 °C, respectively, while these values for the cyclic polymer were 27 °C and 35 °C, respectively, indicating that the LCST and CP temperature of the cyclic polymer are lower [8]. CP is a macroscopic parameter that defines the temperature at which a solution becomes turbid and can be confirmed by visual inspection. On the other hand, the LCST is defined as a point on the phase diagram and is measured by critical temperature measurement using DCS measurement [11,12]. Here, the onset temperature at which the transmittance decreases by 1% can be regarded as the transition temperature from coil-to-sphere, so the values at which the transmittance changes by 1% and 50% were used in this study to compare with the results reported by Liu et al. [8]. Since it is known that the temperature response of polyNIPAM depends on the molecular weight of the polymer [35], we synthesized cyclic-polyNIPAM_360_ (*M*_n_ = 28,000, *M*_w_/*M*_n_ = 1.53) and linear-polyNIPAM_450_ (*M*_n_ = 36,000, *M*_w_/*M*_n_ = 1.61) with relatively similar molecular weight and molecular weight distribution. Due to the broad molecular weight distribution of the cyclic-polyNIPAM_360_ as prepared by RE-RAFT polymerization, the polymer sample was fractionated by preparative SEC.

**Figure 5:**
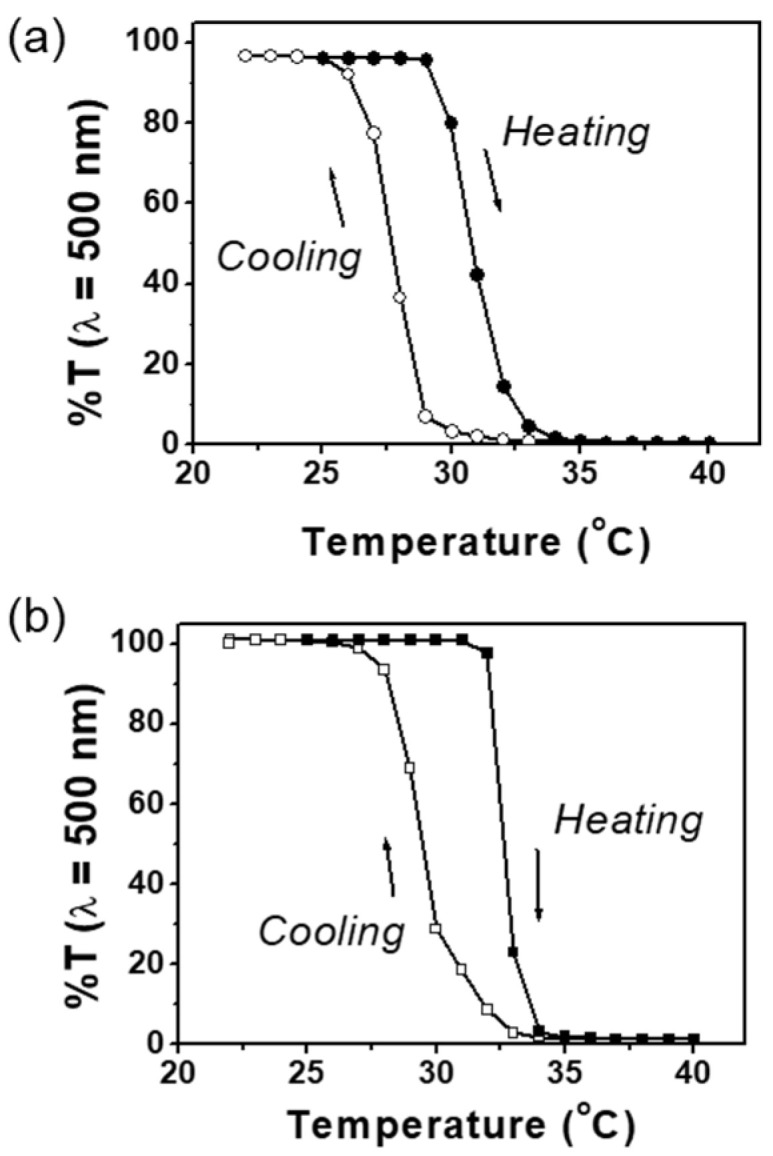
Temperature dependences of optical transmittance at 500 nm obtained for 1.0 g/L aqueous solutions of (**a**) cyclic-polyNIPAM_360_ and (**b**) linear-polyNIPAM_450_.

Figure 5 shows the temperature dependence of the transmittance of aqueous solutions (concentration of 1.0 g/L) of cyclic-polyNIPAM_360_ (Figure 5a) and linear-polyNIPAM_450_ (Figure 5b) at a wavelength of 500 nm upon heating and cooling at a rate of 0.5 °C/min. Upon heating the aqueous solutions, the linear-polyNIPAM_450_ solution showed a rapid decrease in transmittance, while the cyclic-polyNIPAM_360_ solution showed a more gradual decrease. Based on the point at which the transmittance of the solutions began to decrease, the onset temperature for the cyclic-polyNIPAM_360_ and the linear-polyNIPAM_450_ were determined to be 29 °C and 32 °C, respectively, indicating that, above these temperatures, interchain interactions began to occur, forming aggregates, which resulted in a decrease in transmittance due to scattering of the incident light. Furthermore, the CP temperatures were determined from the temperature at which transmittance reaches 50%, which were 31 °C and 33 °C for the cyclic-polyNIPAM_360_ and the linear-polyNIPAM_450_, respectively. For these temperatures, the cyclic-polyNIPAM_360_ exhibited lower temperatures than the linear-polyNIPAM_450_. Thus, during the temperature increase process, the water molecules that were hydrated to the polyNIPAM segments dehydrated at a relatively low temperature to form intramolecular hydrogen bonds, resulting in a lower onset temperature for the cyclic-polyNIPAM_360_. This result is similar to the trend previously reported for the thermo-responsive properties of the cyclic- and linear-polyNIPAMs by Liu [8]. On the other hand, as the temperature decreased, the linear-polyNIPAM_450_ began to rapidly increase in transmittance, while the cyclic-polyNIPAM_360_ showed a clearly slower rise in transmittance and remained opaque until around 30 °C. It is speculated that these phenomena are because the cyclic-polyNIPAM has no terminal structure, which makes the molecular chains more constrained compared to the linear analog, allowing the formation of enhanced intramolecular hydrogen bonds. That is, during the temperature decrease process, the intramolecular hydrogen bonds were formed relatively strongly in the cyclic-polyNIPAM, making it difficult for redissolution due to hydration to occur.

## 3. Materials and Methods

### 3.1. Chemicals and Reagents

Unless otherwise stated, all commercial reagents including 2,2′-azobis(4-methoxy-2,4-dimethylvaleronitrile) (V-70; FUJIFILM Wako Pure Chemical Corporation, Osaka, Japan, 95%) and *S*,*S*-dibenzyl trithiocarbonate (DBTTC; FUJIFILM Wako Pure Chemical Corporation, Osaka, Japan, 98%) were used as received. *N*-isopropylacrylamide (NIPAM; FUJIFILM Wako Pure Chemical Corporation, Osaka, Japan, 98%) was recrystallized from a mixture of toluene/*n*-hexane (1/1, *v*/*v*) prior to use. A cyclic trithiocarbonate derivative, 4,7-diphenyl-[1,3]dithiepane-2-thione (CTTC) was synthesized according to the literature [36,37].

### 3.2. Methods

^1^H NMR spectra were assessed in methanol-*d*_4_ or DMSO-*d*_6_ at 25 °C on a Bruker model AC-500 spectrometer (Bruker, Billerica, MA, USA), operating at 500 MHz, where chemical shifts (*δ* in ppm) were determined with respect to non-deuterated solvent residues as internal standards. Analytical size exclusion chromatography (SEC) was performed at 40 °C, using 8.0 mm × 300 mm polystyrene gel columns (TOSOH-TSKgel H type × 2) on a TOSOH GPC-8320 system (TOSOH, Tokyo, Japan), equipped with a UV-8000 variable-wavelength UV–vis detector (TOSOH, Tokyo, Japan). The number-average molecular weight (*M*_n_) and molecular weight distribution (*M*_w_/*M*_n_) were calculated from the chromatographs with respect to 15 polystyrene standards (Scientific Polymer Products, Inc., Ontario, NY, USA; *M*_n_ = 580–670,000 g mol^−1^, *M*_w_/*M*_n_ = 1.01–1.07). Preparative size exclusion chromatography (SEC) was performed at 25 °C by using 21.5 mm × 300 mm polystyrene gel columns (TOSOH TSKgel G2000H, G2500H, and G3000H) on a TOSOH model CCPE equipped with a RI-8022 RI detector (TOSOH, Tokyo, Japan). Matrix-assisted laser desorption/ionization time-of-flight mass spectrometry (MALDI-TOF-MS) was performed on a Bruker model amaZon SL spectrometer (Bruker, Billerica, MA, USA) using THF as a solvent, 9-nitroanthracene as a matrix, and sodium trifluoroacetate as a cationizing agent. UV–vis spectra were recorded using a quartz cell of 1 cm path length on a JASCO Type V-730 spectrometer equipped with a Peltier thermostatted cell holder ETCS-761 (JASCO, Hachioji, Japan).

### 3.3. Ring-Expansion RAFT Polymerization of NIPAM with CTTC (Synthesis of Cyclic-polyNIPAM_30_, Cyclic-polyNIPAM_52_ and Cyclic-polyNIPAM_122_)

Ring-expansion RAFT polymerization of NIPAM was carried out with CTTC as a cyclic RAFT agent and V-70 as an initiator. To a solution of NIPAM (321 mg, 2.8 mmol) and CTTC (4.5 mg, 14 μmol) in acetonitrile (1.28 g) was added V-70 (1.9 mg, 6.0 μmol) in a glass tube ([NIPAM]_0_/[CTTC]_0_/[V-70]_0_ = 200/1/0.4). The resulting solution was degassed by three freeze-pump-thaw cycles, and then the glass tube was sealed under vacuum, heated at 60 °C for 0.5–2 h, and quenched by rapid cooling. The reaction mixture was analyzed by SEC and ^1^H NMR spectroscopy. By the ^1^H NMR spectra of reaction mixtures, the NIPAM conversions were calculated based on the peak intensity ratio of the isopropyl methyl protons and the vinyl protons of NIPAM. The acetonitrile solution of the reaction mixture was poured into a large amount of *n*-hexane to precipitate the polymers and remove the unreacted monomers. The resultant polymer was collected by centrifugation and dried under reduced pressure. The isolated polymer structure of cyclic-polyNIPAM_122_ was analyzed by ^1^H NMR spectroscopy and the isolated polymer of cyclic-polyNIPAM_30_ was analyzed by MALDI-TOF-MS.

### 3.4. Conventional RAFT Polymerization of NIPAM with DBTTC (Synthesis of Linear-polyNIPAM_146_)

Conventional RAFT polymerization of NIPAM was carried out with DBTTC as a bifunctional acyclic RAFT agent and V-70 as an initiator. To a solution of NIPAM (161 mg, 1.4 mmol) and DBTTC (2.1 mg, 7.2 μmol) in acetonitrile (642 mg) was added V-70 (0.86 mg, 2.8 μmol) in a glass tube ([NIPAM]_0_/[DBTTC]_0_/[V-70]_0_ = 200/1/0.4). The resulting solution was degassed by three freeze-pump-thaw cycles, and then the glass tube was sealed under vacuum, heated at 60 °C for 0.5–2 h, and quenched by rapid cooling. The reaction mixture was analyzed by SEC and ^1^H NMR spectroscopy. The acetonitrile solution of the reaction mixture was poured into a large amount of *n*-hexane to precipitate the polymers and remove the unreacted monomers. The resultant polymer was collected by centrifugation and dried under reduced pressure. The isolated polymer structure was analyzed by ^1^H NMR spectroscopy and MALDI-TOF-MS.

### 3.5. Synthesis of Cyclic-polyNIPAM_360_ and Linear-polyNIPAM_450_ for Analysis of Temperature-Responsive Properties

For the synthesis of cyclic-polyNIPAM_360_, V-70 (0.76 mg, 2.4 μmol) was added to a solution of NIPAM (321 mg, 2.8 mmol) and CTTC (1.8 mg, 5.6 μmol) in acetonitrile (1.28 g) in a glass tube ([NIPAM]_0_/[CTTC]_0_/[V-70]_0_ = 500/1/0.4). The resulting solution was degassed by three freeze-pump-thaw cycles, and then the glass tube was sealed under vacuum, heated at 60 °C for 2 h, and quenched by rapid cooling. The acetonitrile solution of the reaction mixture was poured into a large amount of *n*-hexane to precipitate the polymers and remove the unreacted monomers. The resultant polymer was collected by centrifugation and dried under reduced pressure. Furthermore, the obtained polymer sample was fractionated by preparative SEC.

For the synthesis of linear-polyNIPAM_450_, V-70 (0.34 mg, 1.1 μmol) was added to a solution of NIPAM (161 mg, 1.4 mmol) and DBTTC (0.84 mg, 2.8 μmol) in acetonitrile (642 mg) in a glass tube ([NIPAM]_0_/[DBTTC]_0_/[V-70]_0_ = 500/1/0.4). The resulting solution was degassed by three freeze-pump-thaw cycles, and then the glass tube was sealed under vacuum, heated at 60 °C for 2 h, and quenched by rapid cooling. The acetonitrile solution of the reaction mixture was poured into a large amount of *n*-hexane to precipitate the polymers and remove the unreacted monomers. The resultant polymer was collected by centrifugation and dried under reduced pressure.

## 4. Conclusions

In this study, we demonstrated that thermo-responsive cyclic-polyNIPAM can be successfully synthesized by ring expansion RAFT polymerization of NIPAM using a cyclic chain transfer agent CTTC. This RE-RAFT polymerization is a smart synthetic method that can efficiently obtain the desired cyclic polymers by simply adding a cyclic RAFT agent to a conventional free radical polymerization system. Since cyclic-polyNIPAM does not possess chain ends, it exhibits a different thermal phase transition behavior compared to linear-polyNIPAM with almost the same molecular weight and molecular weight distribution, exhibiting lower onset and CP temperatures. It is speculated that the cyclic topology facilitates intramolecular hydrogen bond formation in cyclic-polyNIPAM. By using the RE-RAFT polymerization, it is possible to synthesize multi-component cyclic polymers by copolymerization of NIPAM with other acrylamide monomers, so it is expected that it can be applied to the control of thermo-responsive properties. Large-scale synthesis of cyclic polymers enable us to elucidate the relationships between the physical and functional properties of cyclic polymers and their structures in detail.

## Figures and Tables

**Table 1:** Molecular weights and molecular weight distributions of the cyclic-polyNIPAMs obtained by RE-RAFT polymerization of NIPAM with a RAFT reagent CTA.

	CTA	Reaction Time (h)	NIPAM Conversion (%) ^a^	*M*_n, theory_^b^ (g mol^−1^)	*M*_n, top_ ^c,d^ (g mol^−1^)	*M*_w_/*M*_n_^e^
cyclic- polyNIPAM_30_	CTTC	0.75	15	3400	6400 and 1300	2.15
cyclic- polyNIPAM_52_	CTTC	1	26	5900	8300	1.68
cyclic- polyNIPAM_122_	CTTC	2	61	14,000	8000	1.78
cyclic- polyNIPAM_360_	CTTC	2 ^e^	72	41,000	28,000	1.53
linear- polyNIPAM_146_	DBTTC	2	73	16,000	6600	1.21
linear- polyNIPAM_450_	DBTTC	2 ^e^	90	51,000	36,000	1.61

^a^ Determined by ^1^H NMR. ^b^ Calculated from *M*_n, theory_ = ([NIPAM]_0_/[CTA]_0_ × (molecular weight of NIPAM) × (conversion of NIPAM). ^c^ Estimated by PSt-calibrated SEC in THF. ^d^ Estimated from peak top value of SEC curve. ^e^ Polymerization conditions; [NIPAM]_0_/[CTA]_0_/[V-70]_0_ = 500/1/0.4, [NIPAM]_0_ = 20 wt%.

## Data Availability

Data are contained within the article.
